# Basketball Players Jump and Maximal Strength Performance Improvements After French Contrast Training

**DOI:** 10.3390/sports14080350

**Published:** 2026-08-12

**Authors:** Yiqing Xie, Yemin Han, Qing Du, Zhen Zhang, Rodrigo Ramirez-Campillo, Kai Xu

**Affiliations:** 1 Sport, Exercise and Health Sciences, Loughborough University, Epinal Way, Loughborough LE11 3TU, Leicestershire, UK; 2221151070@sus.edu.cn; 2 School of Athletic Performance, Shanghai University of Sport, No. 200, Henren Road, Shanghai 200438, China; 3 Exercise and Rehabilitation Sciences Institute, Faculty of Rehabilitation Sciences, Universidad Andres Bello, Santiago 405, Chile; 4 Department of Physical Activity Sciences, Universidad de Los Lagos, Osorno 1046, Chile; 5 School of Medical and Health Sciences, Centre for Human Performance, Edith Cowan University, Perth 6027, Australia

**Keywords:** plyometric exercise, muscle strength, human physical conditioning, resistance training, ball sports

## Abstract

(1) Background: French contrast training (FCT) enhances sports performance, but its long-term efficacy in elite basketball players remains unclear. This study compared FCT and traditional strength training (TST) on jump performance and lower-limb strength. (2) Methods: Sixteen elite basketball players (age 22.4 ± 1.4 years; 14 male, 2 female) were randomly assigned to 8 weeks of FCT (n = 8) or TST (n = 8). The TST protocol consisted of barbell back squat, barbell Bulgarian split squat, conventional deadlift, and barbell hip thrust (4 sets of 8, 6, 4, 4 repetitions at 80%, 95%, 90%, 90% of one-repetition maximum [1RM]; 2–3 min inter-set rest). Assessments included countermovement jump (CMJ) kinetics, CMJ with arm swing (CMJA), running CMJ, running single-leg CMJ, and back squat 1RM, analyzed via repeated-measures ANOVA. (3) Results: A significant Group × Time interaction favored FCT for maximal relative force (*p* = 0.007, ηp^2^ = 0.41): FCT increased 6.80% (Cohen’s d = 1.55), TST decreased 2.41% (d = −0.36). Significant time effects (*p* < 0.001–0.044) occurred for CMJA, running CMJ, flight height, reactive strength index, and back squat 1RM, with large improvements in both groups (e.g., back squat 1RM: FCT +30.45%, d = 2.89; TST +26.77%, d = 2.34). The interaction for average power approached significance (*p* = 0.053, ηp^2^ = 0.24). No other interactions were significant. (4) Conclusions: Both FCT and TST improved performance, but FCT yielded superior gains in maximal relative force. Limitations include small sample (n = 16), mixed-gender composition, short 8-week intervention, and lack of retention testing.

## 1. Introduction

Competitive basketball demands high-paced aerial play, involving frequent and varied jumping actions (e.g., shooting, rebounding, blocking) that are multi-directional, rapidly repeated, and performed under competitive conditions, along with aerial confrontation and high-intensity physical collisions [1]. Therefore, competitive basketball players require a combination of key physical and physiological qualities, including high-level strength, power, speed, agility, metabolic conditioning, and motor control [2]. Indeed, vertical jump performance significantly influences the flow of competition and is a key factor in determining offensive and defensive outcomes [3]. In addition to jumping performance, maximal strength plays a role in basketball performance. For example, higher relative BS 1RM strength levels are associated with enhanced performance in basketball-critical abilities—specifically vertical jump height and change-of-direction speed [4]. Greater strength measures (higher relative BS 1RM) in elite academy basketball players were correlated with improved faster acceleration, better agility, and higher jump reach [5]. Stronger center players (BS 1RM > 2.0 × body mass vs. <2.0 × body mass) exhibited a 22% higher rebound rate per 40 min of elite basketball play, and stronger guard players (BS 1RM ≥ 1.8 × body mass vs. <1.8 × body mass) showed a 31% greater steal efficiency per 40 min of play [6].

To develop these qualities, traditional strength training (TST)—typically comprising multi-joint resistance exercises with progressive overload—has been widely adopted in basketball conditioning programs. However, alternative methods such as French contrast training (FCT) may offer additional benefits by integrating strength, plyometric, and velocity-based stimuli within a single session. To improve vertical jump and maximal strength performance, a common intervention involves FCT [7]. This training system combines elements of complex and contrast training [8]. The core sequence of FCT typically includes: (i) heavy-load multi-joint resistance exercises (e.g., back squat at 80–90% of 1 repetition maximum [RM]); (ii) bodyweight plyometric exercises (e.g., depth jumps or hurdle hops); (iii) velocity-based training using reduced loads (e.g., hex bar jumps at ~30% 1RM); and (iv) assisted eccentric-isometric training using external aids (e.g., band-assisted jumps) [9]. This multi-phase protocol may enhance resistance to power decrements during fatiguing conditions. This is particularly relevant for basketball, as match-play induces significant and persistent reductions in vertical jump performance [10]. Supportively, a study in trained individuals demonstrated maintained countermovement jump height following fatigue when using this protocol compared to traditional training [11]. FCT is recognized for its acute enhancement of jump performance, attributed to mechanisms such as increased neural drive, tendon stiffness, activation of fast-twitch fibers, and reduced neural inhibition [12]. Its efficient structure makes it particularly suitable for athletes with limited training time [3]. For instance, an acute 8.5% increase in CMJ height was observed following an FCT session compared to TST in recreational athletes [9], while an even greater acute improvement of 12.3% in CMJ height after FCT versus 3.1% after TST was reported in elite soccer players [13].

It is important to distinguish FCT from simpler post-activation potentiation (PAP) protocols, which typically involve a single heavy resistance exercise followed by a ballistic movement [12]. While PAP methods are less resource-intensive and easier to implement, they provide a narrower loading spectrum. In contrast, FCT exposes athletes to four distinct loading conditions (heavy, plyometric, light-loaded, and assisted) within each set, potentially inducing more comprehensive neuromuscular stimulation across the force–velocity continuum [9,14]. However, this complexity also requires greater equipment availability and coaching supervision. Previous studies comparing contrast training (similar to FCT) with simple PAP have reported mixed findings, with some suggesting superior acute effects of multi-phase protocols [11,13] and others finding comparable improvements [15]. This distinction is relevant for practitioners seeking time-efficient yet effective training strategies.

Beyond acute effects, FCT may induce superior long-term adaptations compared to TST due to its inherent contrast structure. The sequential pairing of heavy resistance (≥85% 1RM) with maximal-velocity ballistic movements is hypothesized to chronically enhance post-activation performance enhancement (PAPE), leading to greater neural drive, improved motor unit recruitment synchronization (especially of high-threshold type II fibers), and increased tendon stiffness over time [12,16,17]. Moreover, the varied loading spectrum within FCT (heavy, plyometric, light-loaded, and assisted) exposes the neuromuscular system to a broader range of force–velocity demands, potentially stimulating more comprehensive neuromuscular adaptations than the predominantly slow-velocity, high-force stimuli of TST [18,19]. These theoretical advantages suggest that FCT could produce greater and more transferable gains in explosive performance than traditional resistance training alone.

Despite these acute benefits, evidence on long-term adaptations remains limited and derives primarily from studies in other sports. For example, after 8 weeks of FCT, female triple-jump athletes showed significant improvements in jump power and technical parameters, though the absence of a control group limits causal inference [20]. Similarly, neuromuscular training, which shares principles with FCT, improved strength and power in adolescent males compared to a flexibility-training control [21]. In elite handball players, 12 weeks of FCT led to gains in bone mineral density, sprint performance, and jump metrics relative to a passive control group [22].

Given the essential role of jump and strength capabilities in elite basketball [3], along with the potential of FCT to enhance such qualities [23], there is a notable gap in controlled long-term research examining FCT specifically in elite basketball players. Therefore, this study aimed to compare the effects of FCT and TST on jump performance and lower-limb maximal strength in this population over a sustained training period. We hypothesized that FCT would elicit greater improvements than TST, based on two lines of reasoning: (i) the acute potentiating effects of contrast loading observed in previous studies [9,13], and (ii) the theoretical advantage of multi-modal stimulation for chronic neuromuscular adaptation [16,17]. Specifically, we predicted that FCT would lead to superior gains in maximal relative force, jump height, and power-related metrics compared to TST.

## 2. Materials and Methods

### 2.1. Participants

Given the small sample (n = 16) and the inclusion of only two female participants without stratification, this study should be considered exploratory, providing preliminary insights rather than generalizable conclusions. Consequently, all inferential analyses described below are exploratory in nature. This study employed a randomized parallel-group design. No protocol modifications occurred post-commencement. The study participants were not involved in the design, conduct, reporting, or dissemination plans of this research. All procedures were conducted at the biomechanics laboratory of Shanghai University of Sport under standardized environmental conditions (22.0 ± 1.0 °C; 55 ± 5% humidity) and approved by the Ethics Committee of the Shanghai University of Sport (Approval No. 102772025RT044), requiring informed signed consent from all participants. The trial protocol was not prospectively registered in a publicly accessible trials registry. Participant enrollment commenced on 20 January 2025.

The sample size was determined (a priori) using G*Power 3.1 [24]. For repeated-measures ANOVA (within-between interaction) with effect size f = 0.25, α = 0.05, and power = 0.80, a total of 28 participants were required to detect significant effects. The final sample consisted of 16 elite athletes, reflecting the practical constraints of recruiting participants meeting the stringent inclusion criteria for this high-performance cohort [25].

The inclusion criteria considered: (i) Current or recent (<2 years) participation in FIBA-recognized Tier 1 leagues [26]; (ii) Ability to lift 1.5× body mass BS 1RM and rhythmic half-squat capacity (5 repetitions ~ 60% body mass in 5 s); (iii) Performance baseline meeting position-specific vertical jump thresholds (>65.0 cm guards, >55.0 cm forwards). The exclusion criteria considered: (i) Recent (<6 months) orthopedic injuries limiting jumping/landing; (ii) Engagement in external resistance/plyometric training (>2 sessions/week); (iii) <90% intervention adherence or inability to meet velocity-based task criteria.

Highly homogeneous elite China University Basketball League (CUBA) Division I athletes (14 males, 2 females; age 22.4 ± 1.4 years; training experience > 8 years; BS 1RM > 1.5 × body mass; coefficient of variation [CV] < 8% in jump performance) were randomly assigned to the FCT (n = 8) or TST (n = 8) group (see Table 1 for baseline characteristics). This smaller-than-planned sample size increases the risk of Type II error [27,28]; therefore, observed effect sizes for all primary outcomes are reported alongside significance testing to aid interpretation. The allocation sequence was computer-generated by an independent statistician who was not involved in participant recruitment or intervention delivery. The randomization used a block randomization procedure with randomly varying block sizes of 4 and 6 to ensure balanced group sizes throughout the trial and to minimize predictability while maintaining a 1:1 allocation ratio. This was a simple randomization without stratification for any covariates (e.g., sex or baseline performance). Sex was not used as a stratification factor due to the study’s primary focus on the training intervention and the limited sample size, which precluded a well-powered analysis of sex-specific effects. We acknowledge that menstrual cycle phase and oral contraceptive use were not controlled for in the two female participants, which represents a limitation given the known effects of hormonal fluctuations on muscle stiffness, ligament laxity, and force production [29,30]. This decision was made due to practical constraints: the recruitment window was limited to a single competitive season, and the small number of female athletes (n = 2) precluded meaningful subgroup analysis or statistical control. However, the sensitivity analysis excluding female participants confirmed the robustness of the primary finding for maximal relative force (*p* = 0.024, ηp^2^ = 0.36), suggesting that the key result was not unduly influenced by female inclusion. Future studies with larger female samples should incorporate menstrual cycle monitoring. Allocation concealment was ensured by using sequentially numbered, opaque, sealed envelopes (SNOSE) prepared by the independent statistician. Enrollment personnel had no access to future allocations. While participants/coaches were unblinded due to intervention distinctiveness, outcome assessors and data analysts remained blinded to group allocation throughout the trial. The same two trained researchers, who were not involved in intervention delivery, conducted all pre- and post-testing. A coding system (unique ID for each participant) was used on all data records. The master list linking IDs to group assignment was kept by the principal investigator and was not disclosed to assessors or analysts until after the final statistical analysis. No unintended unblinding occurred. To minimize expectation bias, all participants received standardized instructions stating that both training methods were evidence-based and equally potentially effective. Coaching staff were directed not to compare the interventions.

### 2.2. Intervention Training Protocol

**Figure 1:**
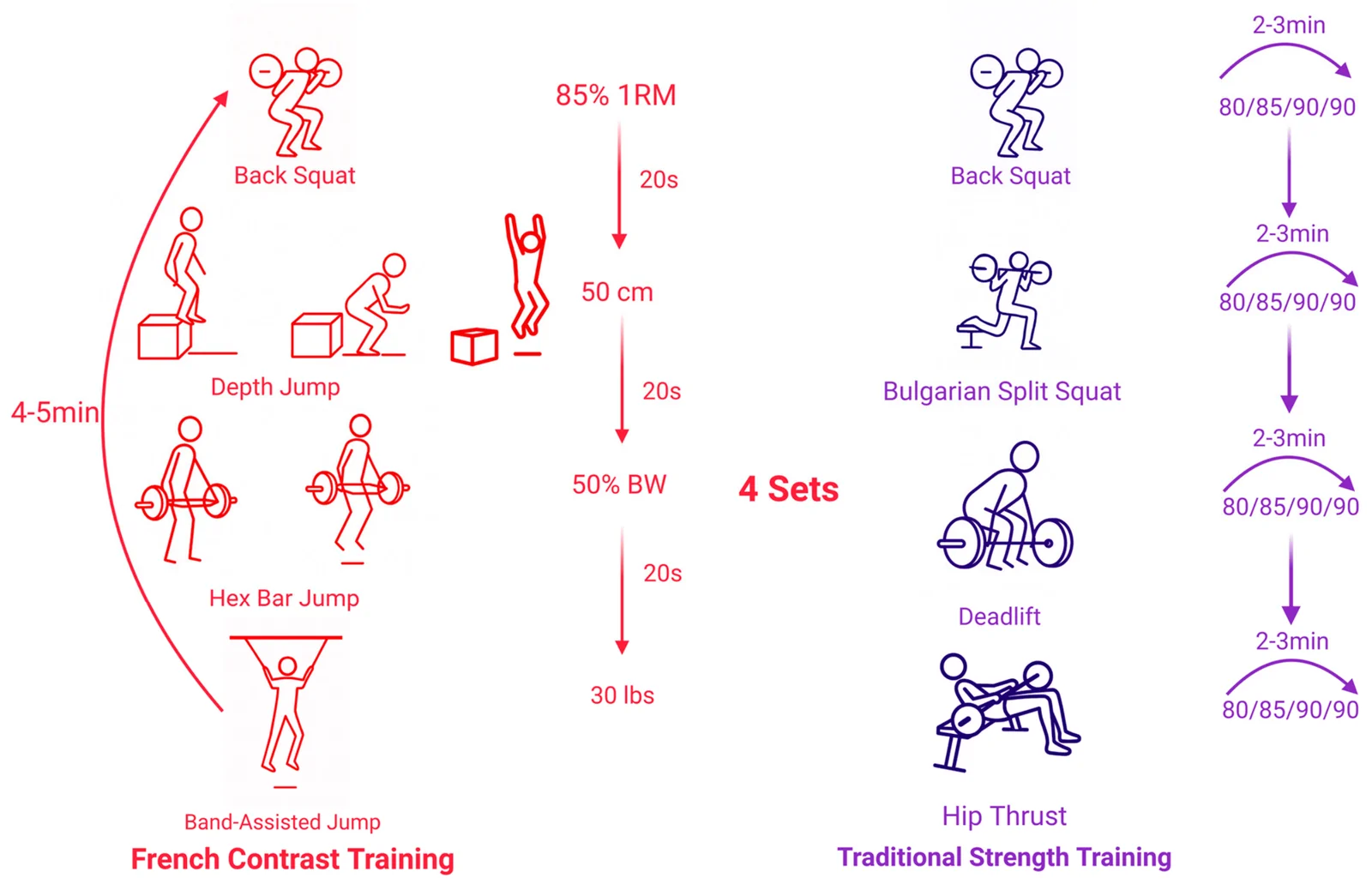
Training protocol for French contrast training (FCT) group and traditional strength training (TST) group.

The intervention was conducted in-season over 8 weeks, with participants completing 6 sessions per week. The weekly schedule included 4 basketball-specific sessions focusing on game-based conditioning, skill development, and tactical execution and 2 dedicated physical conditioning sessions. The experimental FCT intervention was implemented within these 2 physical conditioning sessions, each lasting 45 min, replacing the participants’ regular conditioning work for the study duration. All sessions were structured to include a warm-up (jogging, dynamic stretching, and movement integration drills) and concluded with post-training static stretching and foam rolling. Figure 1 and Table 2 provide details for the FCT and TST protocols. Briefly, the FCT comprised four exercises performed in sequence within each set: (i) barbell back squats at 85% 1RM to maximize neural drive and high-threshold motor unit recruitment [16]; (ii) depth jumps from a 50 cm box to enhance stretch-shortening cycle efficiency and reactive strength [9]; (iii) hex bar jumps at 50% body mass to train ballistic force production at moderate loads [18]; and (iv) elastic band-assisted jumps (13 kg assistance) to promote supra-maximal velocity and reduce neural inhibition [12]. Each set included all four exercises, with rest intervals of ≤20 s. A between-sets rest period of 4–5 min was implemented. The TST involved barbell back squats, followed (always in the same sequence) by barbell Bulgarian split squats, conventional deadlifts, and barbell hip thrusts [31]. These exercises were selected to target maximal lower-body strength through multi-joint compound movements (back squat), unilateral stability and strength (Bulgarian split squat), posterior chain development (deadlift), and glute-dominant hip extension (hip thrust) [31]. Sets were separated by 2–3 min of rest. Of note, the total volume load (sets × repetitions × load) for the barbell back squat differed between protocols: FCT = 13.6 × 1RM per session; TST = 19.3 × 1RM per session. Rest intervals also varied (≤20 s within FCT complexes vs. 2–3 min between TST sets). These differences confound the comparison between training modalities, and this issue is addressed in the Limitations. Furthermore, session rating of perceived exertion (session-RPE) was not collected, which is a limitation. Before their inclusion in the study, all the participants had ≥1 year of consistent TST experience, showing proficiency in performing the required training movements. Therefore, the TST group was considered the control group in the study design, while the experimental group replaced TST with FCT. Intervention sessions were monitored by certified strength coaches (CSCS) with ≥3 years working in elite basketball. To quantify internal training load, heart rate was monitored throughout all sessions using chest-strap telemetry (Polar H10, Polar Electro Oy, Kempele, Finland). Intensity zones were defined as percentages of age-predicted maximum heart rate (HRmax = 220 − age): Zone 1 (light, 50–69% HRmax), Zone 2 (moderate, 70–89% HRmax), and Zone 3 (hard, 90–100% HRmax). Mean exercise heart rate and time in these intensity zones were analyzed, confirming that all sessions were executed within prescribed ranges without excessive cardiovascular strain. Heart rate is an indirect and limited indicator of neuromuscular demand; session rating of perceived exertion (sRPE) or velocity-based tracking would have been more valid. However, sRPE was not collected due to coaching staff workload constraints, and velocity-based devices were unavailable. This limitation is further discussed in Section 4.4. All participants belonged to the same university team (CUBA Division I) and completed the same 4 basketball-specific sessions per week together, including team practices, tactical drills, and scrimmages. Official match participation was similarly distributed between groups, as all players were part of the same roster and playing time was determined by coaching staff based on performance and positional needs, independent of group assignment. Therefore, external training and match loads were considered comparable between the FCT and TST groups. However, individual external load (e.g., minutes played, practice volume) was not systematically quantified, which is acknowledged as a limitation. Adverse events were systematically recorded per session via structured logs, defining injuries requiring training modification/cessation or medical attention.

### 2.3. Outcomes

#### 2.3.1. Kinetic Metrics from Countermovement Jump (CMJ)

Following 2–3 familiarization trials, subjects executed the bilateral CMJ from a natural stance, involving a preparatory squat followed by a maximal vertical jump on command. Two official trials were performed under two conditions: with arm swing and without arm swing. Between-trial rest was ≥30 s, with ≥2 min between different test types. The best performance (jump height in meters, recorded to two decimal places) from the two trials for each condition was used for analysis [14].

Kinetic metrics were sampled at 2000 Hz using a force platform (Kistler 9287CA, Kistler Instrumente AG, Winterthur, Switzerland), interfaced with MARS Software v4.3. Raw force signals were filtered using a fourth-order, zero-lag Butterworth low-pass filter with a cut-off frequency of 50 Hz. The initiation of the countermovement was defined as the instant when the vertical ground reaction force deviated more than 5 standard deviations (5 SD) from the subject’s body weight during quiet standing (averaged over 1 s prior to movement onset). The start of the concentric phase was identified as the point where the vertical force exceeded body weight after reaching the lowest center of mass position. Take-off was defined as the instant when the vertical force fell below 10 N. Landing was identified as the instant when the vertical force exceeded 10 N after the flight phase. Flight time was calculated as the interval between take-off and landing, and jump height was derived from flight time using the formula: height = (g × t^2^)/8, where g = 9.81 m/s^2^. All data processing was performed using MARS Software v4.3. The kinetic metrics were selected based on previous studies [16,17], and included: (i) CMJ relative maximal force: Maximum force output during countermovement jump normalized to body mass [6]; (ii) CMJ relative peak power: Peak power output normalized to body mass (W/kg) [32]; (iii) CMJ flight height: Vertical displacement of the body’s center of mass during the airborne phase, correlated to measures of neuromuscular capability [33]; (iv) CMJ reactive strength index (RSI): Metric quantifying reactive force production efficiency during the stretch-shortening cycle, determined by elastic energy utilization and neural pre-activation [34]; (v) CMJ average power: Time-averaged mechanical power output (W) over the entire concentric phase, calculated as total work done/concentric duration, indicating sustainable production efficiency [35]; (vi) CMJ peak power: Maximal instantaneous mechanical power output (W) generated by the neuromuscular system within the movement cycle, reflecting lower-extremity explosive force production efficiency [36]; (vii) CMJ concentric impulse: Time-integral of net vertical force during the propulsive phase, governing takeoff velocity and jump height through force-time accumulation [37]; (viii) CMJ peak concentric velocity: Maximal center-of-mass velocity attained during the concentric phase [38]; (ix) CMJ take-off velocity: Instantaneous center-of-mass velocity at ground contact termination, correlated to jump height in elite athletes [18].

#### 2.3.2. Field-Based CMJ with Arm-Swing (CMJA) Performance

Four field-based CMJA performance metrics were selected for assessment based on their relevance to basketball-specific jump demands, encompassing static, dynamic, and unilateral movement scenarios [16,17,39]. The specific tests were administered as follows: (i) CMJA. Maximal vertical jump height from a static, bipedal stance was evaluated using a standardized protocol [40]. Participants began standing with feet parallel under a calibrated jump mat, performed a self-selected countermovement, and then executed a maximal effort vertical jump with a full arm swing. Jump height was determined via flight time. Participants performed three trials, and the best result was recorded [41]. (ii) Running CMJ. Vertical jump height following a horizontal approach was assessed to replicate a common basketball maneuver [42]. Participants initiated the jump from a self-selected 6–10 m approach run, took off from both legs simultaneously for maximal height, and jump height was measured using a jump mat. The peak height from three valid attempts was used for analysis. (iii) Running Single-Leg CMJ. Unilateral explosive power under dynamic conditions was assessed using a running single-leg CMJ performed with the dominant leg, a method applied in sports performance testing [43]. The protocol involved a self-selected 6–10 m approach, with take-off and landing on the same leg. The highest jump from three valid attempts was recorded. (iv) Back Squat One-Repetition Maximum (BS 1RM). The BS 1RM was assessed following a standardized testing protocol to ensure pre- and post-test consistency [14,44]. Testing sessions were scheduled at the same time of day (±2 h) for each participant [45], with a minimum of 72 h of rest after the last training session to ensure full recovery [46]. All sessions began with a standardized dynamic warm-up. The testing protocol involved progressive loading: after initial warm-up sets, participants performed 3–5 maximal attempts with 3–5 min of rest, aiming to reach the 1RM within 5 attempts [47]. Squat depth was strictly controlled (top of thigh at hip below knee level) and technique was monitored in real-time by a certified specialist; only repetitions meeting the depth and technique criteria (neutral spine, proper knee tracking) were accepted [48]. The highest successfully lifted load was recorded as the 1RM.

Field-based CMJA performance tests were conducted using a Vertec^®^ Vertical Jump Trainer (Sports Imports, Worthington, OH, USA) set at a standard height of 3.05 m. Each subject completed the jump protocols after performing two warm-up attempts at 50% and 70% of perceived maximum effort. Each test involved attempts separated by ≥2 min, and different test categories were separated by ≥5 min [14,49]. The best performance, recorded as maximal touch height (the highest vane displaced, in centimeters), was used for analysis.

### 2.4. Statistical Analysis

All analyses were conducted in R (version 4.3.2; R Core Team, 2023) using the following packages: tidyverse (version 2.0.0) [50] for data manipulation, rstatix (version 1.1.0) [51] for ANOVA analyses, emmeans (version 2.0.4) [52] for post hoc comparisons, effectsize (version 1.0.0) [53] for effect size calculations, and pwr (version 1.3–0) [54] for power analysis. The primary analysis followed the intention-to-treat principle, including all 16 randomized participants. No post-randomization exclusions occurred, and the dataset was complete. Assumptions of normality (Shapiro–Wilk test on residuals) and homogeneity of variances (Levene’s test) were met for all variables. Baseline characteristics are presented descriptively (Table 1). Reliability of key outcomes was assessed via intraclass correlation coefficient (ICC) using a two-way mixed-effects model for absolute agreement (ICC(3,1); [55]), with 95% CIs computed via bootstrapping (2000 replicates). The analysis demonstrated excellent reliability metrics, with ICCs ranging from 0.92 to 0.97. In accordance with conventional benchmarks, ICC values greater than 0.90 are considered to indicate excellent reliability [55]. The analytical approach included: (i) Primary analysis: 2 × 2 repeated-measures ANOVA (Group × Time) to test main and interaction effects [56]. Simple main effects analyses were conducted for significant or trending interactions (*p* < 0.05). (ii) Sensitivity analysis: (a) ANCOVA models with post-intervention scores as dependent variables, Group as a fixed factor, and baseline scores as covariates [57]; and (b) as a secondary sensitivity analysis, we re-ran all primary analyses on the male-only subsample (n = 14) to examine the consistency of the findings after excluding the two female participants. Because the trial was not prospectively registered, no outcome was designated a priori as primary. All analyses are thus exploratory, and *p*-values are unadjusted for multiplicity. To reduce false-positive risk, we emphasize effect sizes (ηp^2^, Cohen’s d) with their 95% confidence intervals rather than relying solely on significance thresholds. Statistically significant findings should be interpreted cautiously. Maximal relative force was of particular interest based on prior literature, but this does not imply it was a prespecified primary outcome.

For all analyses, we report F-values, degrees of freedom, exact *p*-values, partial eta-squared (ηp^2^) with 95% confidence intervals, and observed power. Effect sizes were interpreted as: ηp^2^ = 0.01 (small), 0.06 (medium), 0.14 (large) [58]. Statistical significance was set at *p* < 0.05.

## 3. Results

### 3.1. Pre-Intervention Equivalence

Data met assumptions for parametric tests (normality of residuals confirmed by Shapiro–Wilk tests, homogeneity of variances by Levene’s tests). Randomization yielded groups that were well-matched on most demographic, anthropometric, and jump performance measures (Table 1). However, a baseline imbalance was observed for strength measures, with the FCT group showing higher values in both absolute and relative back-squat 1RM compared to the TST group. To control for these pre-existing differences in the analysis of training adaptations, all between-group comparisons on post-intervention outcomes were performed using analysis of covariance (ANCOVA), adjusting for their respective baseline values, in addition to the primary repeated-measures ANOVA.

### 3.2. Vertical Jump Performance

Repeated measures ANOVA revealed no statistically significant Group × Time interactions for any of the vertical jump measures (CMJA: F(1,14) = 0.65, *p* = 0.434, ηp^2^ = 0.04 [small], observed power = 0.13; Running CMJ: F(1,14) = 3.05, *p* = 0.102, ηp^2^ = 0.18 [large], observed power = 0.41; Running single-leg CMJ: F(1,14) = 1.70, *p* = 0.214, ηp^2^ = 0.11 [medium], observed power = 0.25). Supportive ANCOVA analyses, adjusting for baseline values, yielded significant between-group differences in post-intervention scores for Running CMJ (F(1,13) = 6.68, *p* = 0.023, ηp^2^ = 0.34 [large]) and single-leg CMJ (F(1,13) = 5.20, *p* = 0.040, ηp^2^ = 0.29 [large]), while the effect for CMJA approached significance (F(1,13) = 4.47, *p* = 0.054, ηp^2^ = 0.26 [large]).

**Figure 2:**
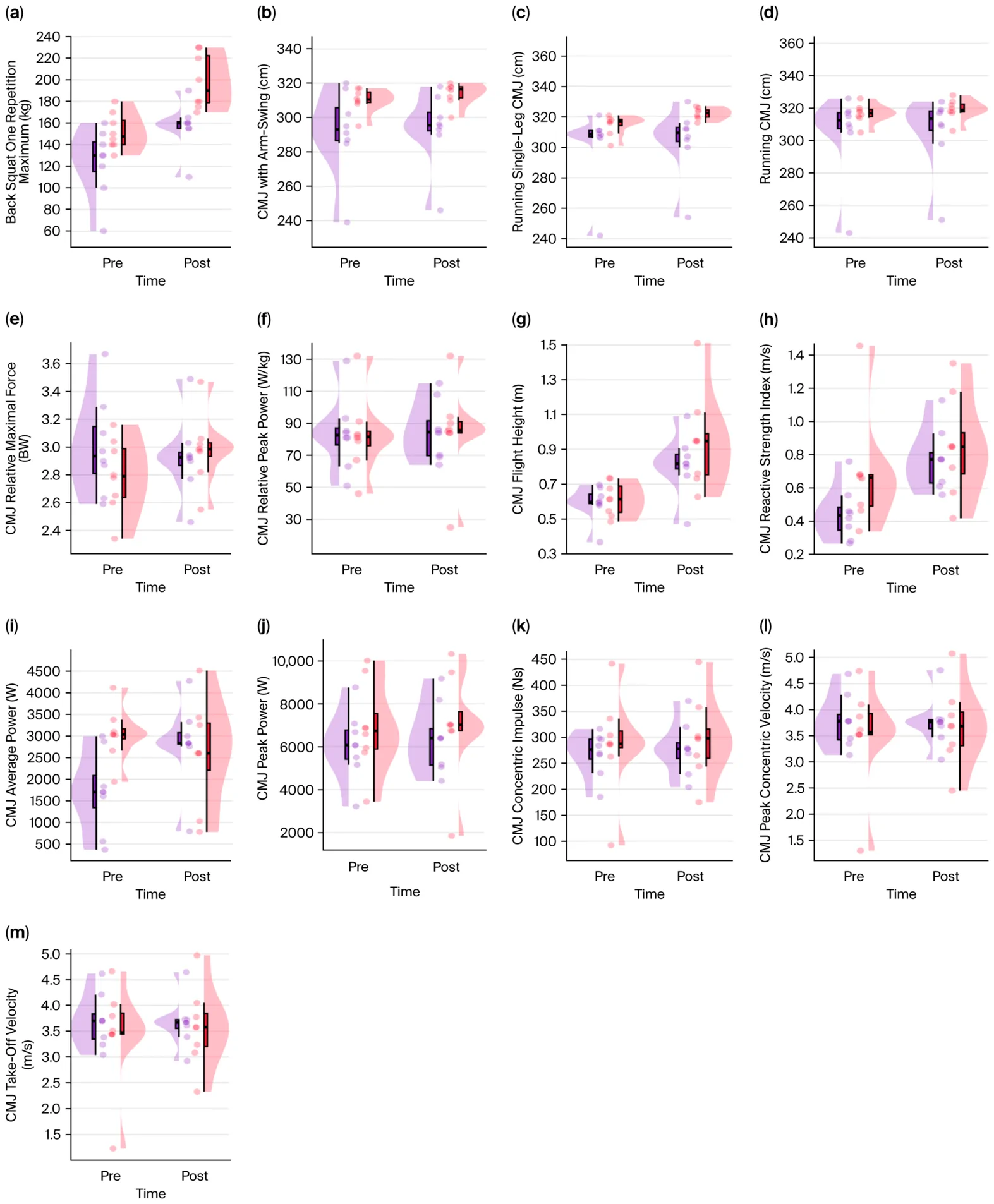
Effects of French contrast training (pink) and traditional strength training (purple) on basketball players’ jump and strength performance. (**a**) Back Squat One Repetition Maximum (kg); (**b**) CMJ with Arm-Swing (cm); (**c**) Running Single-Leg CM (cm)J; (**d**) Running CMJ (cm); (**e**) CMJ Relative Maximal Force (BW); (**f**) CMJ Relative Peak Power (W/kg); (**g**) CMJ Flight Height (m); (**h**) CMJ Reactive Strength Index (m/s); (**i**) CMJ Average Power (W); (**j**) CMJ Peak Power (W); (**k**) CMJ Concentric Impulse (Ns); (**l**) CMJ Peak Concentric Velocity (m/s); (**m**) CMJ Take-Off Velocity (m/s).

A significant main effect of Time was observed for CMJA (F(1,14) = 4.91, *p* = 0.044, ηp^2^ = 0.26 [large]). Descriptively, the FCT group showed a 1.21% increase from pre- to post-test (Cohen’s d = 1.50 [large]), while the TST group improved by 0.60% (d = 0.27 [small]) (Figure 2). For running CMJ, a significant main effect of Time was also found (F(1,14) = 13.54, *p* = 0.002, ηp^2^ = 0.49 [large]), with the FCT group demonstrating a 2.34% improvement (d = 1.65 [large]) compared to a 0.87% gain in the TST group (d = 0.42 [small]). The Group × Time interaction approached significance (*p* = 0.102, observed power = 0.41). No significant main effect of Time was found for running single-leg CMJ (F(1,14) = 3.82, *p* = 0.071, ηp^2^ = 0.21 [large]).

### 3.3. Kinetic and Kinematic Adaptations to Jumping

A significant Group × Time interaction was identified for maximal relative force in both RM-ANOVA (F(1,14) = 9.90, *p* = 0.007, ηp^2^ = 0.41 [large], 95% CI [0.09, 0.73], observed power = 0.88) and ANCOVA (F(1,13) = 6.70, *p* = 0.022, ηp^2^ = 0.34 [large]) analyses. This finding remained robust in a sensitivity analysis excluding female participants (F(1,12) = 6.70, *p* = 0.024, ηp^2^ = 0.36 [large]). Post hoc analysis indicated divergent responses: the FCT group significantly increased maximal relative force by 6.80% (Cohen’s d = 1.55 [large]), whereas the TST group showed a decrease of −2.41% (d = −0.36 [small]).

A strong main effect of Time was observed for flight height (F(1,14) = 16.03, *p* = 0.001, ηp^2^ = 0.53 [large], 95% CI [0.21, 0.80]), with large improvements in both the FCT (54.31%, d = 1.05 [large]) and TST groups (37.47%, d = 0.97 [large]). The ANCOVA for flight height was not significant (F(1,13) = 1.80, *p* = 0.202, ηp^2^ = 0.12 [medium]). The interaction was non-significant (F(1,14) = 0.65, *p* = 0.434, observed power = 0.13).

For the RSI, significant main effects were found for both Time (F(1,14) = 5.07, *p* = 0.041, ηp^2^ = 0.27 [large]) and Group (F(1,14) = 5.50, *p* = 0.034, ηp^2^ = 0.28 [large]). Large within-group effect sizes were observed (TST: d = 1.24 [large]; FCT: d = 0.30 [small]). The ANCOVA for RSI was not significant (F(1,13) = 2.00, *p* = 0.181, ηp^2^ = 0.13 [medium]). The interaction was non-significant (F(1,14) = 0.53, *p* = 0.479, ηp^2^ = 0.04 [small], observed power = 0.11).

The Group × Time interaction for mean power approached significance in RM-ANOVA (F(1,14) = 4.45, *p* = 0.053, ηp^2^ = 0.24 [large], observed power = 0.55) but not in ANCOVA (F(1,13) = 0.03, *p* = 0.863, ηp^2^ < 0.01 [small]). Descriptively, the TST group exhibited a large improvement (66.05%, d = 0.71 [medium]), while the FCT group showed a small decline (−14.34%, d = −0.32 [small]).

No significant interactions or main effects of Time were found for concentric impulse (F(1,14) = 0.00, *p* = 0.959, observed power = 0.05), concentric peak velocity (F(1,14) = 0.50, *p* = 0.490, observed power = 0.11), takeoff velocity (F(1,14) = 0.40, *p* = 0.536, observed power = 0.10), peak power (F(1,14) = 0.10, *p* = 0.760, observed power = 0.06), or relative peak power (F(1,14) = 0.00, *p* = 0.945, observed power = 0.05) (all *p* > 0.05).

### 3.4. Maximal Strength Adaptations

For BS 1RM, there were significant main effects of Time (F(1,14) = 110.80, *p* < 0.001, ηp^2^ = 0.89 [large], 95% CI [0.79, 0.95]) and Group (F(1,14) = 8.82, *p* = 0.010, ηp^2^ = 0.39 [large], 95% CI [0.07, 0.72]) in RM-ANOVA. The ANCOVA confirmed a significant between-group difference after adjusting for baseline (F(1,13) = 5.64, *p* = 0.034, ηp^2^ = 0.30 [large]). Both groups achieved large strength gains (FCT: 30.45%, d = 2.89 [large]; TST: 26.77%, d = 2.34 [large]) (Table 3). The Group × Time interaction was non-significant (F(1,14) = 3.03, *p* = 0.104, ηp^2^ = 0.18 [large], observed power = 0.41).

### 3.5. Statistical Power Considerations

The observed statistical power for detecting Group × Time interactions varied considerably across variables. Only maximal relative force achieved adequate power (power = 0.88). For all other variables, power was insufficient to reliably detect interaction effects, increasing the risk of Type II error (false negatives). Specifically, the observed power values were as follows: CMJA (0.13), running CMJ (0.41), running single-leg CMJ (0.25), flight height (0.13), reactive strength index (0.11), mean power (0.55), concentric impulse (0.05), concentric peak velocity (0.11), takeoff velocity (0.10), peak power (0.06), relative peak power (0.05), and back squat 1RM (0.41). Readers should interpret non-significant interaction effects with caution, as the study was underpowered for these comparisons. Power to detect main effects of Time was adequate (≥0.80) for variables with large effect sizes (ηp^2^ > 0.45), including running CMJ (0.95), flight height (0.98), and BS 1RM (1.00).

## 4. Discussion

The primary aim of this study was to compare the effects of an 8-week FCT intervention against TST on basketball-specific jump performance and kinetic adaptations in elite collegiate players. The key findings indicate that while both training modalities were effective for improving overall lower-body performance, FCT elicited a distinct and favorable adaptation in the force production domain. Specifically, FCT led to a clear increase in maximal relative force during jumping, whereas a decrease was observed in the TST group. Both groups demonstrated significant improvements in various jump height measures and maximal strength. However, no other differential adaptations between the training protocols reached statistical significance.

Baseline imbalance in strength measures (Table 1) raises the possibility of a ceiling effect, whereby the stronger FCT group might have had less room for improvement. Despite this constraint, FCT still demonstrated a significant advantage in maximal relative force, while the weaker TST group did not exhibit larger gains. Thus, the interaction favoring FCT is unlikely to be driven solely by baseline differences. Nonetheless, future studies should employ stratified randomization to better control for such confounders.

### 4.1. Jump Performance Adaptations

Both groups demonstrated significant improvements in vertical jump performance, as indicated by the main effects of Time for CMJA and running CMJ. This finding aligns with previous research that reported contrast training’s effectiveness for reactive jump performance [15]. The primary differential adaptation, however, was observed in maximal relative force, which showed a significant Group × Time interaction (*p* = 0.007, ηp^2^ = 0.41). This result supports the original hypothesis, providing preliminary evidence for FCT’s unique efficacy in enhancing force-production efficiency—a quality critical for basketball-specific movements like explosive rebounding and blocking [3]. However, it must be noted that this was the only variable for which statistical power was adequate (power = 0.88); for all other interaction effects, power ranged from 0.05 to 0.55, meaning that non-significant results do not necessarily imply absence of effect but may reflect insufficient sample size. In a game context, this improved force capability is crucial for a player to quickly generate the necessary leg drive for a second or third consecutive jump during intense rebounding sequences under fatigue. The observed advantage for FCT is at the level of the integrated training program. While the program’s design incorporates the principle of contrast loading (sequentially pairing heavy squats with maximal-effort jumps), which is theorized to enhance the rate of force development through mechanical specificity and chronic neuromuscular adaptations [16,19], the present comparison of two multi-faceted training programs does not isolate this component. Therefore, mechanistic attributions to specific elements like PAP remain plausible but hypothetical.

In contrast, the lack of a significant Group × Time interaction for concentric impulse suggests that the chronic translation of this specificity into improved impulse was not statistically confirmed. Furthermore, the absence of differential improvements in metrics like flight height may be attributed to multiple factors. The 8-week intervention duration might be insufficient for the full consolidation of distinct chronic neuromuscular adaptations that directly translate to enhanced net performance outcomes [16,17]. Additionally, the absence of dedicated jump technique training, which focuses on movement coordination and efficiency, may have limited the expression of improved force capabilities into greater jump height, as the intervention primarily targeted physical adaptations rather than skill refinement. The principle of training specificity suggests that the adaptations elicited by FCT and TST, while beneficial for force and power, may not have been sufficiently specific to the complex coordinative demands of maximizing flight height. Consequently, a longer intervention period may allow FCT’s superior adaptation in force production to translate into performance differences. Furthermore, the substantial difference in training volume between protocols (FCT squat volume load: 13.6 × 1RM vs. TST: 19.3 × 1RM per session) and rest intervals (≤20 s vs. 2–3 min) means that the observed effects cannot be unequivocally attributed to the specific nature of FCT, as volume and density are confounded with the intervention type. Overall, the findings confirm that FCT specifically optimizes the force component of the force-velocity profile, the biomechanical foundation for high-force movements. This is speculatively attributed to the protocol’s strategic sequencing, which may exploit chronic PAPE mechanisms and trade-offs over time [16,17,59]. It is noteworthy that the RSI showed a large increase in the TST group despite its non-plyometric design. This may be partially attributable to the higher baseline jumping proficiency in the FCT group, leaving less room for improvement, and highlights that RSI can be influenced by factors beyond a direct plyometric stimulus, such as improvements in concentric force production or landing strategy learned during training components.

To further contextualize the specific benefits of FCT, it is useful to compare it with other common methods. Simple PAP protocols (single heavy exercise followed by a ballistic movement) are less resource-intensive but provide a narrow loading stimulus, primarily targeting neural drive without addressing the full force–velocity spectrum [12]. Complex training pairs a heavy exercise with a biomechanically similar plyometric movement, offering a broader stimulus than PAP but lacking FCT’s velocity-based and assisted phases [8]. Traditional contrast training alternates heavy and light loads across sets but typically omits FCT’s four-phase sequence [9]. In contrast, FCT integrates four distinct loading conditions (heavy resistance, plyometric, light-loaded ballistic, and assisted eccentric-isometric) within each set, exposing the neuromuscular system to a wider force–velocity range in a single session [9,16]. This multi-modal approach may benefit time-limited athletes requiring simultaneous strength, power, and reactive development. However, FCT demands greater equipment (boxes, hex bars, bands) and coaching expertise, limiting applicability in less-resourced settings. Method selection should therefore consider the athlete’s training history, available equipment, and performance goals.

### 4.2. Force, Velocity, and Power Adaptations

The present study provides specific evidence that FCT induced a statistically superior adaptation in maximal relative force (significant Group × Time interaction, *p* = 0.007), supporting the original hypothesis of its efficacy. However, given the low statistical power for most other variables (power = 0.05–0.55) and the aforementioned volume load disparity, this finding should be interpreted with caution. For other kinetic and kinematic variables (e.g., take-off velocity, peak power), the analysis did not yield statistically significant Group × Time interactions (*p* > 0.05). However, descriptive trends within the FCT data conceptually align with prior research reporting the benefits of contrast training for explosive qualities [18,60]. The trend towards improved velocity and power metrics, if consolidated over time, could translate to faster second-effort plays, such as a quick put-back attempt after an offensive rebound. From a practical perspective, it is noteworthy that the FCT program achieved its effects on force with a lower total repetition volume for the core lower-body compound lift compared to TST, potentially offering a time-efficient training strategy.

Mechanistically, the contrast structure within FCT is theorized to elicit adaptation through dual-phase potentiation. High-intensity squats are proposed to induce transient PAP, while subsequent ballistic jumps exploit this state to enhance motor unit firing frequency and reflex sensitivity, optimizing neural drive during the velocity-dependent concentric phase [61]. The ≤4 min inter-set rest is designed to coincide with peak potentiation while managing fatigue. This structure mirrors basketball’s intermittent demands, and the principle of pairing heavy loads (>85% 1RM) with ballistic actions is well-supported for velocity adaptation [18,62], supporting the rationale behind FCT’s underlying design. The absence of statistically significant interactions for most velocity-power metrics, despite these mechanistic premises and positive trends, may be attributed to the 8-week intervention. This duration may have been sufficient for the clear force adaptation observed, but potentially insufficient for the full consolidation of differentiated neuromuscular adaptations in more complex outcomes. Furthermore, the divergent magnitudes of change among power metrics (e.g., mean vs. peak power) likely reflect their calculation from different segments of the force-time curve. The substantial increase in mean concentric power for TST may indicate an improvement in the ability to sustain power output throughout the entire concentric phase, a quality distinct from peak instantaneous power. Future research with longer durations is needed to determine if these acute physiological effects translate into statistically robust, long-term improvements across the full spectrum of performance variables.

### 4.3. Strength Enhancements

Both FCT and TST elicited significant improvements in BS 1RM, as evidenced by a strong main effect of Time (*p* < 0.001). The statistical analysis did not yield a significant Group interaction for this variable (*p* = 0.104), indicating that the magnitude of strength gain over 8 weeks was not statistically different between the two training modalities. This finding aligns with previous research that observed both complex and traditional training can improve maximal strength, though potentially through distinct adaptive pathways [22]. The similar strength gains occurred despite the lower squat volume in FCT, underscoring its potential efficiency. However, given the baseline imbalance (FCT group started stronger) and the low power for the interaction (power = 0.41), a true difference cannot be ruled out. Enhanced maximal lower-body strength directly supports in-game physicality, such as maintaining position during a box-out or holding ground in a post-up situation against an opponent.

The neurophysiological rationale underpinning FCT proposes that the sequenced exposure to high-load resistance followed by maximal-velocity jumps is designed to optimize the neural and contractile properties for explosive force production. This sequence may potentiate motor unit recruitment synchronization (particularly of high-threshold type II fibers), enhance inter-muscular coordination, and accelerate cross-bridge cycling kinetics, thereby augmenting the rate of force development during explosive concentric actions [63]. While the present study’s primary analysis did not confirm a differential effect on 1RM, the observed descriptive trend showing a greater mean improvement in the FCT group, combined with its specific positive effect on maximal relative force (*p* = 0.007), suggests that its structure may favorably influence the neuromuscular efficiency of force expression. Conversely, TST’s emphasis on maximal loads may predominantly stimulate morphological adaptations and neural drive suited to slower velocities, which is reflected in the non-significant descriptive trends for velocity-related metrics and aligns with the concept that velocity-specific adaptations require corresponding high-velocity stimuli. The lack of a significant Group × Time interaction for the RSI further indicates that 8 weeks may be insufficient to induce differentiated, long-term adaptations in stretch-shortening cycle capabilities, which are known to require sustained or distinct eccentric-overload stimuli [64]. In summary, while both training methods effectively increased maximal strength, the hypothesized superiority of FCT in translating strength gains into a neuromuscular profile favoring high-velocity force production was primarily observed for maximal relative force. The observed dissociation between the large improvement in 1RM and the slight decrease in platform-derived maximal relative force for the TST group underscores that these metrics capture distinct neuromuscular qualities. The 1RM assesses maximal voluntary strength under slower velocities, while the force platform metric reflects peak force output during a fast, ballistic action. This pattern suggests enhanced slow-velocity maximal strength without translating to improved peak force in a dynamic jump. The intervention duration may have prioritized acute neuromuscular efficiency over differentiated long-term morphological adaptations, highlighting the importance of timeframes in interpreting training-specific outcomes.

### 4.4. Potential Limitations

Several limitations warrant consideration. First, while an a priori power analysis indicated 28 participants were required, this study enrolled 16 elite athletes—a sample constrained by the exclusivity of the high-performance cohort. The primary constraint of this smaller, albeit highly homogeneous (CV < 8% for jump metrics), sample is on the external validity of the findings, limiting their generalizability. The randomized design protects internal validity, and the large observed effect sizes for key outcomes partially offset the limited N, an approach recognized in elite sport research [26,65]. Nevertheless, the final sample size remains below common recommendations, which limits statistical power, increases the risk of Type II error, and can result in less stable effect size estimates [27,28]. Second, the mixed-gender sample, while reflecting team composition, meant sex-stratified analysis was not performed; however, sensitivity analyses excluding female participants supported the stability of the key finding for maximal relative force (*p* = 0.024), suggesting the primary result was not unduly confounded by sex. Nevertheless, the inclusion of only two female participants without stratification by sex is a methodological limitation, as it precludes a well-powered analysis of sex-specific effects and limits the generalizability of the findings to female athletes [29,30]. Furthermore, menstrual cycle phase and oral contraceptive use were not controlled for in the female participants, which is a significant confounding factor given that hormonal fluctuations can affect muscle stiffness, ligament laxity, and neuromuscular performance [29,30]. This limitation should be addressed in future research by including adequate numbers of female athletes with standardized hormonal profiling. Third, the exclusive focus on elite CUBA basketball players severely limits the generalizability of the findings, which cannot be extrapolated to adolescents, recreational players, or athletes at sub-elite levels [66,67]. Fourth, the 8-week intervention duration, while effective for eliciting neuromuscular improvements, may be insufficient for inducing longer-term morphological adaptations in structures like tendons, which are critical for the stretch-shortening cycle and can require 10–12 weeks for measurable changes in stiffness [68]. The absence of a retention test further means the study assessed only immediate effects, not the persistence of adaptations over time [69,70]. Fifth, variations in participants’ uncontrolled, club-based daily training could have introduced confounding variables related to cumulative fatigue and recovery, potentially modulating individual responses to the intervention [71,72]. Future studies should implement systematic monitoring of training load and athlete status (e.g., via session-RPE and wellness questionnaires) to control for these extraneous influences, as such measures are known to correlate with performance outcomes and readiness in team sports [73]. Sixth, the statistical analysis employed separate repeated-measures ANOVA and ANCOVA models. Although ANCOVA effectively controlled for the observed baseline imbalances in strength measures in the present study, a unified linear model incorporating baseline scores as a covariate could offer a more flexible framework for future research, particularly in trials with more complex designs or missing data. Seventh, internal training load was monitored solely via heart rate, which is a notoriously poor metric for quantifying the neuromuscular and mechanical demands of high-intensity resistance and plyometric training. Heart rate primarily reflects cardiovascular stress and does not capture the specific neural and muscular fatigue induced by heavy resistance exercises (e.g., back squats at 85–90% 1RM) or explosive ballistic movements (e.g., depth jumps, hex bar jumps). Validated metrics for resistance training, such as sRPE or velocity-based tracking, were not utilized. sRPE was not collected due to logistical constraints during the in-season competitive period, where coaching staff prioritized other monitoring responsibilities. Velocity-based devices were not available for all training sessions. This is a significant methodological flaw, as it limits our ability to equate the internal training load between the FCT and TST protocols and to determine whether differences in neuromuscular fatigue contributed to the observed adaptations. Future studies should prioritize the use of sRPE and/or velocity-based monitoring to provide a more comprehensive assessment of internal training load in contrast training studies. Eighth, the FCT and TST protocols differed substantially in training volume and rest intervals, which confounds the comparison between the two modalities. Specifically, the volume load (sets × repetitions × load) for the barbell back squat was 13.6 × 1RM per session in FCT versus 19.3 × 1RM per session in TST, and rest intervals within FCT complexes were ≤20 s compared to 2–3 min between TST sets. These inherent differences make it difficult to determine whether the observed effects are attributable to the specific nature of FCT or simply to differences in training volume and density. Future studies should consider matching volume load or employing a dose-equivalent design to isolate the specific effect of the contrast method. Ninth, individual external loads from basketball sessions and games were not monitored (e.g., via accelerometry or minute logging). Although both groups shared the same training environment, unmeasured differences in playing time or practice intensity could have influenced adaptations. Future studies should incorporate wearable technology to quantify external load. Tenth, the marked disparity between one athlete’s exceptional standing CMJ (1.5 m) and his relatively modest running CMJ highlights task-specific neuromuscular adaptations, underscoring the need for multi-modal jump assessments in future research. Finally, while PAPE is the hypothesized mechanism for FCT, this study did not include direct physiological measures to confirm its chronic role, highlighting a need for future mechanistic inquiry.

### 4.5. Practical Applications

The following practical suggestions are offered with explicit differentiation between evidence levels.

Procedures used in the present study. Our intervention employed a specific FCT protocol: heavy back squat (87–90% 1RM) → depth jump → hex bar jump → band-assisted jump, performed twice per week for six weeks. Inter-exercise rest was ≤20 s, with ≥3–4 min between sets, and total volume was limited to 4 sets of 4 repetitions per exercise. Athletes in this study possessed a relative back squat ≥1.5× body mass and prior plyometric experience. These parameters were directly tested and may serve as a reference for practitioners working with similarly trained populations.

Recommendations supported by external literature. The use of contrast training complexes to exploit PAPE is well documented in the literature [9]. The general principle of scheduling high-intensity sessions at least 48 h before competition is consistent with recovery guidelines for team-sport athletes [10]. Monitoring readiness via session-RPE or CMJ-derived metrics is a widely adopted practice [73].

Speculative coaching considerations. Several suggestions—such as replacing the final band-assisted jump with a basketball-specific movement (e.g., running rebound jump), individualizing box height or band tension based on athlete characteristics, or reverting to simpler PAP/complex training if fatigue accumulates—are logical extensions of the underlying principles but were not directly tested in this study. Coaches should apply these ideas cautiously, pilot them within their own context, and adjust based on individual athlete responses.

## 5. Conclusions

This study provides preliminary, exploratory evidence that both FCT and TST may improve lower-limb strength and vertical jump performance in elite basketball players over 8 weeks. FCT produced a greater improvement in maximal relative force than TST in this small exploratory sample. Most other outcomes improved over time without clear evidence of differential effects between interventions. However, these findings must be interpreted with extreme caution due to: (i) low statistical power for most interactions (0.05–0.55); (ii) substantial differences in training volume and rest intervals between protocols; (iii) baseline strength imbalances; (iv) the very small sample (n = 16, including only 2 females, which precludes any meaningful sex-specific analysis); and (v) the exclusive focus on elite CUBA players, which limits generalizability to other populations. Therefore, these results should be considered hypothesis-generating rather than confirmatory. Future research with adequately powered samples, matched training volumes, stratified randomization by sex and baseline strength, and longer intervention periods is necessary to validate these preliminary observations.

## Figures and Tables

**Table 1:** Baseline characteristics of participants.

Variable	Overall Sample (n = 16)	FCT Group (n = 8)	TST Group (n = 8)
**Demographics**			
Age (years)	22.4 ± 1.4	22.3 ± 1.5	22.5 ± 1.3
Sex (Male/Female)	14/2	7/1	7/1
Height (cm)	192.8 ± 5.2	192.1 ± 5.4	193.5 ± 5.0
Body Mass (kg)	87.2 ± 4.7	87.2 ± 4.9	87.2 ± 4.5
BMI (kg/m^2^)	23.4 ± 1.0	23.4 ± 1.1	23.4 ± 0.9
**Training Background**			
Training Experience (years)	8.9 ± 0.7	8.8 ± 0.8	9.0 ± 0.6
**Baseline Performance**			
Back Squat 1RM (kg)	137.8 ± 28.5	151.9 ± 16.9	123.8 ± 31.6
Relative Back Squat 1RM	1.58 ± 0.10	1.74 ± 0.11	1.42 ± 0.09
CMJA Height (cm)	301.1 ± 20.6	310.1 ± 7.0	292.0 ± 25.1
Running Single-Leg CMJ Height (cm)	310.9 ± 20.9	316.6 ± 6.0	305.3 ± 26.0
Running CMJ Height (cm)	308.3 ± 20.3	314.8 ± 6.6	301.9 ± 24.6

Note. Data are presented as Mean ± Standard Deviation or frequency (count). BMI, body mass index; 1RM, one-repetition maximum; CMJ, countermovement jump; CMJA, CMJ with arm-swing. CMJA Height, Running Single-Leg CMJ Height, and Running CMJ Height values (in cm) represent the absolute height of the highest vane touched on the Vertec^®^ device.

**Table 2:** Training intervention protocols.

**French contrast training exercises ●**
Barbell back squat, 85% 1RM
Depth jump, 50 cm box
Hex bar jump, 50% body mass
Elastic band-assisted jump, 13 kg
Traditional strength training exercises *
Barbell back squat
Barbell Bulgarian split squat
Conventional deadlift
Barbell hip thrust

●: Exercises involved 4 sets, 4 repetitions/set, with 20 s of inter-set rest. *: Exercises involved 4 sets of 8, 6, 4, and 4 repetitions at 80%, 95%, 90%, and 90% of one repetition maximum (1RM; respectively), with 2–3 min of inter-set rest.

**Table 3:** Summary of Statistical Analyses for All Performance Variables.

Variable	FCT Pre (M ± SD)	FCT Post (M ± SD)	FCT Δ%	FCT d [95% CI]	TST Pre (M ± SD)	TST Post (M ± SD)	TST Δ%	TST d [95% CI]	F(1,14)	*p* (G × T)	ηp^2^ [95% CI]	Obs.Pwr	ANCOVA Adj.Diff% [95% CI]	ANCOVA *p*
VERTICAL JUMP														
CMJA	310.13 ± 7.02	313.88 ± 6.51	1.21% *	1.50 [0.28, 2.72]	292.00 ± 25.08	293.75 ± 21.59	0.60% *	0.27 [−0.58, 1.12]	0.65	0.434	0.04 [0.00, 0.41]	0.13	1.74% [−0.06%, 3.55%]	0.054
Running single-leg CMJ	316.63 ± 6.00	319.13 ± 6.45	0.79%	2.70 [0.90, 4.50]	305.25 ± 26.00	305.75 ± 23.52	0.16%	0.12 [−0.72, 0.96]	1.7	0.214	0.11 [0.00, 0.50]	0.25	1.00% [0.08%, 1.92%]	0.04
Running CMJ	314.75 ± 6.56	322.13 ± 3.76	2.34% **	1.65 [0.37, 2.93]	301.88 ± 24.64	304.50 ± 22.27	0.87% **	0.42 [−0.45, 1.29]	3.05	0.102	0.18 [0.00, 0.57]	0.41	2.22% [0.42%, 4.02%]	0.023
CMJ KINETICS														
Max Relative Force	2.79 ± 0.26	2.98 ± 0.26	6.80% **	1.55 [0.31, 2.79]	3.00 ± 0.35	2.93 ± 0.29	−2.41%	–0.36 [−1.22, 0.50]	9.89	0.007	0.41 [0.09, 0.73]	0.88	7.16% [1.35%, 12.97%]	0.022
Concentric Impulse	287.25 ± 96.60	298.13 ± 79.61	3.79%	0.33 [−0.53, 1.19]	268.25 ± 42.26	278.25 ± 50.97	3.73%	0.29 [−0.56, 1.14]	0	0.959	0.00 [0.00, 0.31]	0.05	1.69% [−10.57%, 13.95%]	0.772
Takeoff Velocity	3.44 ± 0.99	3.57 ± 0.77	3.82%	0.29 [−0.56, 1.15]	3.70 ± 0.52	3.67 ± 0.48	−0.80%	–0.05 [−0.89, 0.78]	0.4	0.536	0.03 [0.00, 0.38]	0.1	1.79% [−11.22%, 14.80%]	0.772
Concentric peak velocity	3.52 ± 0.99	3.69 ± 0.76	4.67%	0.36 [−0.50, 1.23]	3.78 ± 0.51	3.76 ± 0.48	−0.44%	–0.03 [−0.87, 0.81]	0.5	0.49	0.04 [0.00, 0.39]	0.11	2.25% [−10.48%, 14.98%]	0.71
Relative Peak Power	82.63 ± 24.23	85.00 ± 29.08	2.87%	0.21 [−0.63, 1.06]	83.38 ± 22.84	85.13 ± 18.33	2.10%	0.08 [−0.76, 0.91]	0	0.945	0.00 [0.00, 0.31]	0.05	0.52% [−22.17%, 23.21%]	0.961
Peak Power	6877.25 ± 2103.05	7026.38 ± 2502.59	2.17%	0.18 [−0.66, 1.02]	6069.00 ± 1599.74	6398.13 ± 1608.82	5.42%	0.23 [−0.61, 1.08]	0.1	0.76	0.01 [0.00, 0.33]	0.06	–2.06% [−22.29%, 18.17%]	0.832
Mean Power	3034.63 ± 612.62	2599.50 ± 1228.55	−14.34%	–0.32 [−1.17, 0.54]	1703.38 ± 936.91	2828.38 ± 964.25	66.05%	0.71 [−0.22, 1.65]	4.45	0.053	0.24 [0.01, 0.62]	0.55	5.63% [−55.22%, 66.48%]	0.863
Reactive Strength Index	0.68 ± 0.34	0.85 ± 0.30	24.62% *	0.30 [−0.56, 1.15]	0.45 ± 0.16	0.77 ± 0.19	73.67% *	1.24 [0.13, 2.36]	0.53	0.479	0.04 [0.00, 0.40]	0.11	31.97% [−14.56%, 78.50%]	0.181
Flight Height	0.61 ± 0.09	0.95 ± 0.27	54.31% **	1.05 [0.01, 2.09]	0.59 ± 0.10	0.81 ± 0.17	37.47% **	0.97 [−0.04, 1.98]	0.65	0.434	0.04 [0.00, 0.41]	0.13	25.20% [−15.08%, 65.48%]	0.202
MAXIMAL STRENGTH														
Back squat 1RM	151.88 ± 16.89	198.13 ± 25.35	30.45% **	2.89 [0.99, 4.80]	123.75 ± 31.59	156.88 ± 22.03	26.77% **	2.34 [0.73, 3.96]	3.03	0.104	0.18 [0.00, 0.57]	0.41	14.36% [2.18%, 26.54%]	0.034

Note. Values are presented as mean ± standard deviation unless otherwise indicated. FCT = French contrast training group; TST = traditional strength training group; CMJ = countermovement jump; Pre = pre-intervention; Post = post-intervention; Δ% = percentage change from pre to post calculated as (Post − Pre)/Pre × 100; d = Cohen’s d for within-group pre-to-post change with its 95% confidence interval (CI); F(1,14) = F-statistic and degrees of freedom for the Group × Time interaction from repeated-measures ANOVA; *p* (G × T) = exact *p*-value for the interaction; ηp^2^ [95% CI] = partial eta-squared with its 95% CI; Obs.Pwr = observed statistical power (post hoc); ANCOVA Adj.Diff% = adjusted between-group difference (FCT minus TST) expressed as a percentage of the overall baseline mean, derived from the analysis of covariance model controlling for baseline values, with its 95% CI; ANCOVA *p* = *p*-value for the group effect in the ANCOVA. Confidence intervals for ηp^2^ and Cohen’s d were computed using the non-central F and non-central t distributions, respectively. * Denotes significant within-group change (*p* < 0.05); ** Denotes significant within-group change (*p* < 0.01).

## Data Availability

The complete dataset generated and analyzed during the current study is available in the Supplementary Information and at the Open Science Framework repository (osf.io/2udtz).

## Supplementary Materials

The following supporting information can be downloaded at: https://www.mdpi.com/article/10.3390/sports14080350/s1.
